# Extirpation of the OS Maxillare Superius

**Published:** 1849-07

**Authors:** 


### EXTIRPATION OF THE OS MAXILLARE SUPERIUS.

The theatre of the Hospital La Pitiè was unusually crowded by anxious students and practitioners, during a recent operation by M. Lisfranc, for the removal of the right superior maxillary bone, in a case of cancer affecting its structure. The first incision commenced at the external commissure of the lips, and extended to the middle of the malar bone. A second was made along the side of the nose, from the lip to the internal angle of the eye, so as not to compromise the lachrymal sac. The quadrilateral flap being raised up, the parts on its inner side, namely, the nose and the middle of the lip, were detached from the subjacent bones, and pushed on one side in order to gain as much light and room as possible. The bone being much softened, the operation could not be performed in the ordinary way. The vault of the palate was divided by the *cisailles,* the use of which M. Listranc prefers to that of the chain-saw, the former instrument acting more rapidly, although the *cisailles,* as M. Lisfranc remarked, made at present are not conveniently handled, and require modification, to which the attention of instrument makers should be directed. The extirpation of the degenerate parts was accomplished by the scissors, the forceps, and the fingers. M. Lisfranc had the surface of the cavity several times felt by different persons, to ascertain if there remained any suspicious portions. The operation was performed with remarkable promptitude and skill, and notwithstanding its unfavorable obstacles, gave rise to no serious hæmorrhage.—*Revue Clinique.*

